# Evaluation of genetic stability in olive callus-induced and meristem-induced shoots using flow cytometry and amplified fragment length polymorphism techniques

**DOI:** 10.1186/s13007-021-00724-7

**Published:** 2021-03-29

**Authors:** Leila Mirzaei, Abbas Yadollahi, Maryam Jafarkhani Kermani, Masoud Naderpour, Ali Asghar Zeinanloo, Maryam Farsi, Dariush Davoodi

**Affiliations:** 1 Department of Horticultural Sciences, Faculty of Agriculture, Tarbiat Modares University (TMU), P. O. Box: 14115-111, Tehran, Iran; 2 Department of Cell and Tissue Culture, Agricultural Research, Education and Extension Organization (AREEO), Agricultural Biotechnology Research Institute of Iran (ABRII), P. O. Box: 31535-1897, Karaj, Iran; 3 Seed and Plant Certification and Registration Research Institute (SPCRI), Agricultural Research, Education and Extension Organization (AREEO), P. O. Box: 31536-1516, Karaj, Iran; 4 Temperate Fruit Research Center, Agricultural Research, Education and Extension Organization (AREEO), Horticultural Research Institute, P. O. Box: 31585-4119, Karaj, Iran

**Keywords:** Genetic stability, AFLP, Periodical mini bioreactor, Light intensity, Carbon source,, Olive (*Olea europaea* L.)

## Abstract

**Background:**

In vitro culture of olive, as an economically valuable tree, has fundamentally a genotype-dependant low micropropagation rate which needs to be improved in already established and newly released cultivars. Various plant tissue culture media, planting systems and growth factors were evaluated in two promissing Iranian olive cultivars ˈAminˈ and ˈMeshkatˈ and the commercial Spanish cultivar ˈArbequinaˈ.

**Results:**

The results showed that cultivars have their specific optimal media, i.e. ˈAminˈ in the MS with 4 mg/L zeatin, ˈArbequinaˈ in the OM with 1 mg/L zeatin, and ˈMeshkatˈ in the OM and MS with 2 mg/L zeatin, which produced significantly a higher number of axillary shoots than other media. The results also indicated a significant improvement in the growth indices of ˈAminˈ (number of axillary shoots) when cultured using periodical mini bioreactor (PMB) in the VS medium. In comparison with VS, OM did not reveal any significant differences when both culturing systems (PMB and semi-solid media (SSM)) were used.

Regarding the effect of carbon source and light intensity, mannitol and 2000 cd sr m^−2^ greatly enhanced ˈArbequinaˈ growth indices (main shoot length and growth quality). The results of genetic stability of callus induced shoots (CIS) and meristem induced shoots (MIS) revealed that 2C DNA value assessed by partec flow cytometery (FCM) had 0.01, 0.03 and 0.08 pg discrepencies in ˈAminˈ, ˈArbequinaˈ and ˈMeshkatˈ, repectively. The Amplified Fragment Length Polymorphism (AFLP) results also indicated that the cultivars were classified regardless of the micropropagation origin (CIS or MIS), except for ˈArbequinaˈ. The AFLP findings showed that ˈArbequinaˈ had the highest dispersal (7–38%) in CIS and MIS, while the Iranian cultivar of ˈMeshkatˈ (5–9%) had the highest stability.

**Conclusions:**

This study indicated the importance of in vitro growth parameters for improving the micropropagation indices of olive cultivars. It showed that optimized protocols (OM, PMB, zeatin, mannitol and 2000 cd sr m^−2^) co-produced larger calli resulting in indirect organogenesis. Based on FCM and AFLP analysis, it can be concluded that true-to-typeness of micropropagated olive was cultivar-dependent.

## Background

Olive (*Olea europaea* L.) is the only edible species belonging to Oleaceae family, which is probably domesticated from subspecies sylvestris [1], and it is amongst the earliest fruit trees in the Middle East [2]. The first olive is cultivated in Asia before 1000 BC. [3]. The genus *Olea* has 30 species all around the world [4] and olive is an outcrossing, diploid species with vast genetic diversity [5]. Due to the multiple applications including oil, wood and fruit, olive is considered an economically valuable tree [2, 6–8].

The in vitro culture of olive is restricted due to the limited sampling season [9], tough cultivar-dependent proliferation [10], slow lateral bud outgrowth [11], mucus accumulation in the intercellular spaces called hyperhydricity [12], inhibitory phenolic compounds [9], costly protocols [11] and high losses of plantlets [9]. Cultivar, media and plant growth regulators (PGRs) are the most critical factors in olive proliferation [13, 14]. Olive growth quality is highly affected by olive media (OM) [10, 15] and zeatin, which is the most effective PGR in shoot regeneration and plays an important role in salvaging the endangered diploid and triploid ˈLaperrineiˈ olive [16]. Culture system also influences olive growth indices [9, 11, 12, 17]. Some olive species are sensitive to be continuously immersed in liquid media due to the severe vitrification [11]. Temporary immersion system (TIS) resolves such problematic issues in tropical crops and it is promising for woody plants [12]. It overcomes gas accumulation, reduces the mass production costs [11] and causes the least hyperhydricity [12]. Survival rates and plantlet quality in TIS is higher than normal solidified media. TIS also economizes plant production by reducing the amount of zeatin (50%) in olive tissue culture as reported in ˈCaninoˈ cultivar [11]. Mannitol, a sugar alcohol consisting of 30% of total carbohydrates in leaves and branches, is the main photosynthesis product in olive [18] and in combination with OM significantly increases shoots and node numbers per shoot [15]. It also uniforms olive secondary axillary shoots [19]. It is also reported that light type is a determinative environmental factor in olive growth [15].

Tissue culture of olive could encounter some physiological abnormalities such as callusing [19]. Although OM normally causes callus in the bottom of petioles and basal part of plantlets, some of the olive cultivars only multiply on OM [9, 20]. Callus formation is mainly dependent on cultivars, media and PGRs [9, 10, 21]. Somaclonal variation can be considered as an approach to breed plants with restricted genetic base, but elite genotypes selection necessitates fidelity during in vitro propagation. Therefore, the detection of genetic uniformity of in vitro-raised plants at an early stage is desirable [22]. Ploidy level stability assessment in olive cultivar ˈsylvestrisˈ multiplied on OM with zeatin after a PGR shock followed by transferring to the same medium without PGRs showed the DNA content reduced 2.5–9% in the micropropagated plants [20]. Intraclonal variations in the quantitative traits of ˈPicualˈ architecture, which are affected by genotype and continual period of in vitro culturing, were reported in the somatically propagated plantlets [23]. Moreover, nuclear DNA content in olive species was significantly different between donor plants and acclimatized plants in highly reproducible histograms [20].

Molecular markers such as AFLP give the chance of early progenies stability check [2, 6, 24]. Because of high discriminating capacity of AFLP and its homogenous distribution in linkage groups, it is a reliable method to investigate olive genetic diversity [8]. AFLP is normally used for map construction due to the huge number of polymorphism and scorable loci [24]. For revealing numerous bands per reaction and valuable number of indices, AFLP is considered the most efficient marker for olive in comparison with SSR and RAPD [24]. In addition, not only the efficiency index was reported the most, but also the average confusion probability was the least [25].

ˈAminˈ and ˈMeshkatˈ (63% and 66% oil, 14 and 21 kg/tree yields, repectively) are two commercial Iranian cultivars with both table and oil consumption which have similar specifications to the commercial ˈArbequinaˈ (65% oil and 25 kg/tree yield in Iran). Therefore, their mass propagation optimization ensuring ascertained genetic trueness is a serious demand. In this study, different plant tissue culture media (OM, MS and VS), planting systems (PMB and SSM) and growth parameters (zeatin concentration, light intensity and carbon source) on 3 cultivars were investigated to obtain the optimized olive propagation protocol. Genetic discrepancy of CIS and MIS was also studied.

## Results

### Effects of zeatin concentration and cultivar on growth indices at proliferation stage

MS containing 4 mg/L zeatin in ˈAminˈ cultivar produced the highest number of axillary shoots, main shoot length, and growth quality (Table 1). There were no negative signs in ˈAminˈ cultivar in high PGR MS. Different PGR concentrations had no impact on ˈArbequinaˈ and there was no distinct trend by increasing the zeatin concentration (Table 1). ˈMeshkatˈ plantlets were depressed by high zeatin concentration (4 mg/L) in both media, though the increment of zeatin only increased callus production as a negative factor (Tables 1 and 3). In ˈMeshkatˈ cultivar, OM and MS containing 2 mg/L zeatin produced well-grown plantlets (Table 1).

**Table 1:** Effects of media and zeatin concentration on growth indices of olive plantlets cv. ˈAminˈ, ˈArbequinaˈ and ˈMeshkatˈ

Growth quality	No. of leaves in main shoot	Main shoot length (cm)	No. of axillary shoots	Zeatin (mg/L)	Medium	Cultivar
2.61 fg	4.16 d	1.40 def	1.33 ab	0	OM	ˈAminˈ
1.09 g	5.64 bcd	0.56 g	0.08 e	1		
1.89 gh	4.81 cd	0.51 g	0.32 de	2		
2.89 efg	5.39 cd	1.34 def	0.33 de	4		
3.36 cdef	7.11 abc	1.03 fg	0.48 cde	0	MS	
3.48 cdef	8.47 ab	1.48 cdef	0.75 bcd	1		
3.12 def	8.96 a	1.48 cdef	0.78 bcd	2		
4.08 abc	9.43 a	2.23 a	1.50 a	4		
4.23 abc	8.57 ab	1.83 abcd	0.17 de	0	OM	ˈArbequinaˈ
4.55 ab	8.71 ab	2.11 ab	1.03 abc	1		
4.67 ab	9.20 a	1.62 bcde	0.75 bcd	2		
4.80 a	9.26 a	2.00 abc	0.77 bcd	4		
3.49 cdef	7.36 abc	1.50 cdef	0.41 cde	0	MS	
2.80 efg	6.61 abcd	1.20 ef	0.11 e	1		
3.73 bcde	8.39 ab	1.41 cde	0.22 de	2		
3.05 def	6.90 abcd	1.51 cdef	0.13 e	4		
4.85 a	6.10 bcd	1.05 fg	0.48 cde	0	OM	ˈMeshkatˈ
4.82 a	6.62 abcd	1.35 def	0.75 bcd	1		
3.74 bcde	7.32 abc	1.43 cde	1.48 a	2		
2.80 efg	3.27 e	1.06 fg	0.14 e	4		
2.88 efg	6.15 bcd	1.22 ef	0.33 de	0	MS	
4.84 a	6.17 bcd	1.64 bcde	0.49 cd	1		
4.90 a	6.93 abcd	1.84 abcd	1.45 ab	2		
3.05 def	3.87 e	1.60 bcde	0.25 de	4		

### Interactive effect of culturing system and genotype at proliferation stage

**Fig. 1:**
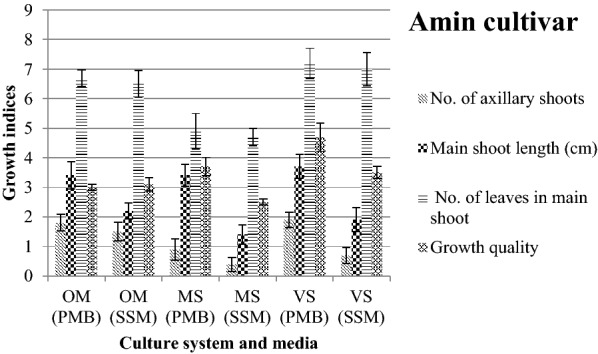
Interaction effects of media and culture system on olive cv ˈAminˈ growth indices. Error bars indicate ± SE

The results showed that most growth indices of ˈAminˈ cultivar were higher in the periodical mini bioreactor (PMB) compared to semi-solid media (SSM) (Fig. 1). In all three culture media PMB system clearly showed the superiority (Fig. 3), but growth quality in SSM and PMB of OM did not show any significant differences (Fig. 1).

**Fig. 2:**
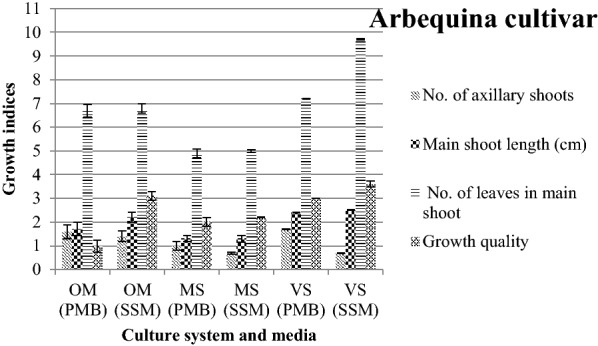
Interaction effects of media and culture system on olive cv.ˈArbequinaˈ growth indices. Error bars indicate ± SE

**Fig. 3:**
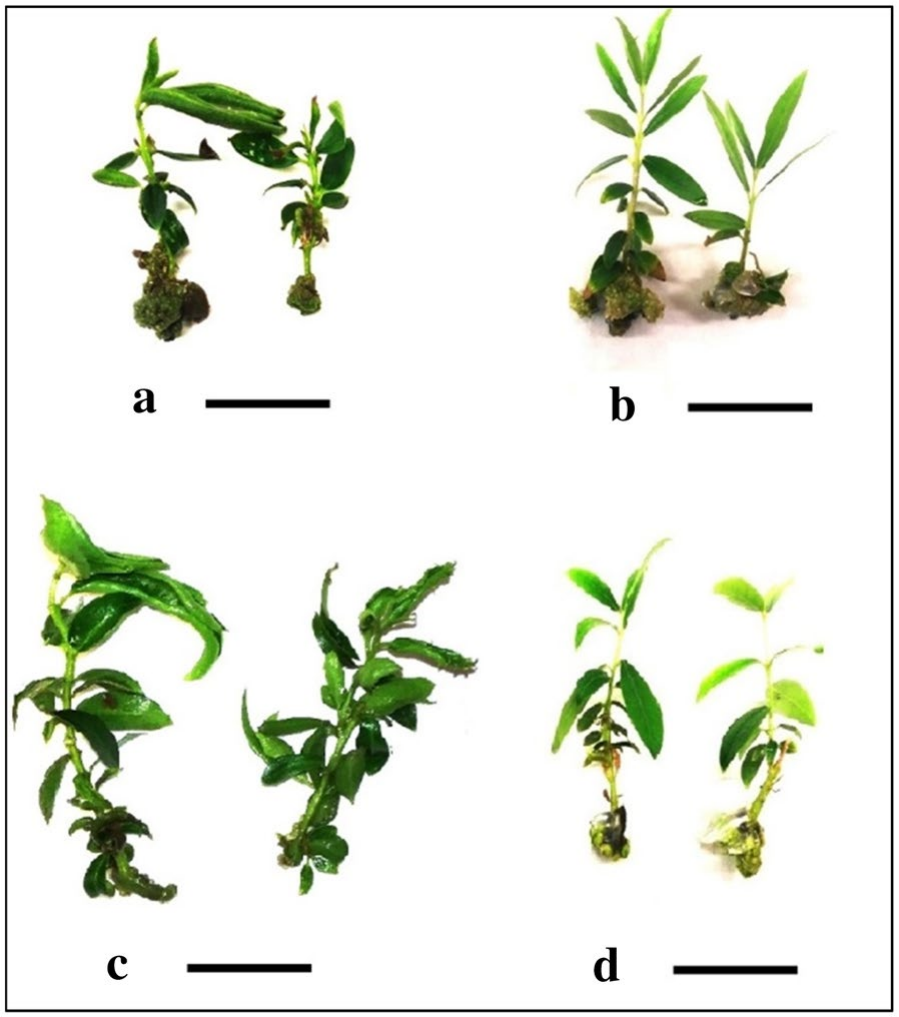
Growth quality, number of axillary shoots, total length and leaves comparison in **a** ˈArbequinaˈ in PMB with VS, **b** ˈArbequinaˈ in SSM with VS, **c** ˈAminˈ in PMB with VS, **d** ˈAminˈ in SSM with VS (scale bar 1.5 cm)

The results showed that among growth indices in ˈArbequinaˈ cultivar only the number of axillary shoots in MS and VS was significantly higher in PMB compared to SSM (Fig. 2). Suffocated plantlets resulted in a reduction in growth quality of plantlets in PMB were compared to SSM (Fig. 3).

Considering OM, main shoot length of ˈAminˈ cultivar increased in PMB, but in ˈArbequinaˈ SSM showed higher growth quality (Figs. 1 and 2). Regarding MS, main shoot length and growth quality in ˈAminˈ cultivar and the number of axillary shoots in ˈArbequinaˈ were higher in PMB than SSM. Regarding VS, it revealed absolute superiority in main shoot length, number of axillary shoots and growth quality using PMB for ˈAminˈ cultivar, however in ˈArbequinaˈ cultivar it did not follow a specific trend (Figs. 1, 2 and 3).

### Carbon and light sources

Main shoot length and growth quality were significantly higher in medium containing mannitol compared to sucrose-enriched medium in ˈArbequinaˈ (Table 2). The results indicated that mannitol and sucrose induced 100% and 81.3% callus initiation at the base of explants, respectively (Table 3). Light intensity at 2000 cd sr m^−2^ enhanced main shoot length, and growth quality of plantlets, whereas the number of axillary shoots and the number of main shoot leaves were not significantly different (Table 2). Callus formation at 2000 cd s m^−2^ (87.5%) was more than 5000 cd sr m^−2^ (75%) (Table 3).

**Table 2:** The simple effect of carbon source and light intensity on ˈArbequinaˈ growth indices (mean ± SE)

Variable	No. of axillary shoots	Main shoot length (cm)	Main shoot leaves	Growth quality
Mannitol	0.9 ± 0.2^a^	3.5 ± 0.1^a^	10.3 ± 0.4^a^	5 ± 0.04^a^
Sucrose	0.7 ± 0.1^a^	2.2 ± 0.19^b^	9 ± 0.6^b^	4.2 ± 0.2^b^
2000 cd sr m-^2^	0.5 ± 0.04 ^a^	3 ± 0.04^a^	9.3 ± 0.21^a^	4.8 ± 0.01^a^
5000 cd sr m-^2^	0.4 ± 0.1^a^	2.1 ± 0.16^b^	9.2 ± 0.5^a^	3.6 ± 0.2^b^

**Table 3:** The effects of treatment and genotype on callus production and volume (mean ± SE)

Cultivar	Medium	Treatment	Callus production (%)	Callus volume (cm^3^)
ˈAminˈ	OM	Zeatin 0 (mg/L)	59.4	1.26 ± 0.12
		Zeatin 1 (mg/L)	57.15	1.52 ± 0.13
		Zeatin 2 (mg/L)	59.4	1.94 ± 0.16
		Zeatin 4 (mg/L)	60.25	2.53 ± 0.21
	MS	Zeatin 0 (mg/L)	87.5	1.89 ± 0.16
		Zeatin 1 (mg/L)	62.5	2.34 ± 0.21
		Zeatin 2 (mg/L)	68.8	2.51 ± 0.17
		Zeatin 4 (mg/L)	30	1.94 ± 0.23
ˈArbequinaˈ	OM	Zeatin 0 (mg/L)	68.75	1.86 ± 0.3
		Zeatin 1 (mg/L)	74.15	2.01 ± 0.26
		Zeatin 2 (mg/L)	75.05	2.09 ± 0.26
		Zeatin 4 (mg/L)	84.4	2.17 ± 0.03
	MS	Zeatin 0 (mg/L)	50	1.83 ± 0.15
		Zeatin 1 (mg/L)	43.8	1.91 ± 0.13
		Zeatin 2 (mg/L)	56.3	2.18 ± 0.12
		Zeatin 4 (mg/L)	68.8	2.56 ± 0.02
ˈMeshkatˈ	OM	Zeatin 0 (mg/L)	15	0.74 ± 0.21
		Zeatin 1 (mg/L)	27.5	1.45 ± 0.34
		Zeatin 2 (mg/L)	35	1.53 ± 0.13
		Zeatin 4 (mg/L)	67.5	2.76 ± 0.41
	MS	Zeatin 0 (mg/L)	10	0.80 ± 0.14
		Zeatin 1 (mg/L)	30	0.80 ± 0.11
		Zeatin 2 (mg/L)	35	0.82 ± 0.07
		Zeatin 4 (mg/L)	65	0.85 ± 0.27
ˈAminˈ	OM + 2 mg/L zeatin	PMB	100	2.25 ± 0.40
		SSM	70	2.03 ± 0.14
	MS + 2 mg/L zeatin	PMB	80	1.44 ± 0.20
		SSM	20	0.12 ± 0.14
	VS + 2 mg/L zeatin	PMB	80	1.19 ± 0.17
		SSM	60	0.9 ± 0.14
ˈArbequinaˈ	OM + 2 mg/L zeatin	PMB	70	2.0 ± 0.26
		SSM	70	1.98 ± 0.24
	MS + 2 mg/L zeatin	PMB	70	3.50 ± 0.11
		SSM	10	3.30 ± 0.23
	VS + 2 mg/L zeatin	PMB	60	3.70 ± 0.17
		SSM	40	1.10 ± 0.12
	OM + 1 mg/L zeatin	Mannitol	100	3.25 ± 0.3
		Sucrose	81.3	1.78 ± 0.28
	OM + 1 mg/L zeatin	2000 cd sr m^−2^	87.5	1.88 ± 0.06
		5000 cd sr m^−2^	75	1.69 ± 0.24

**Fig. 4:**
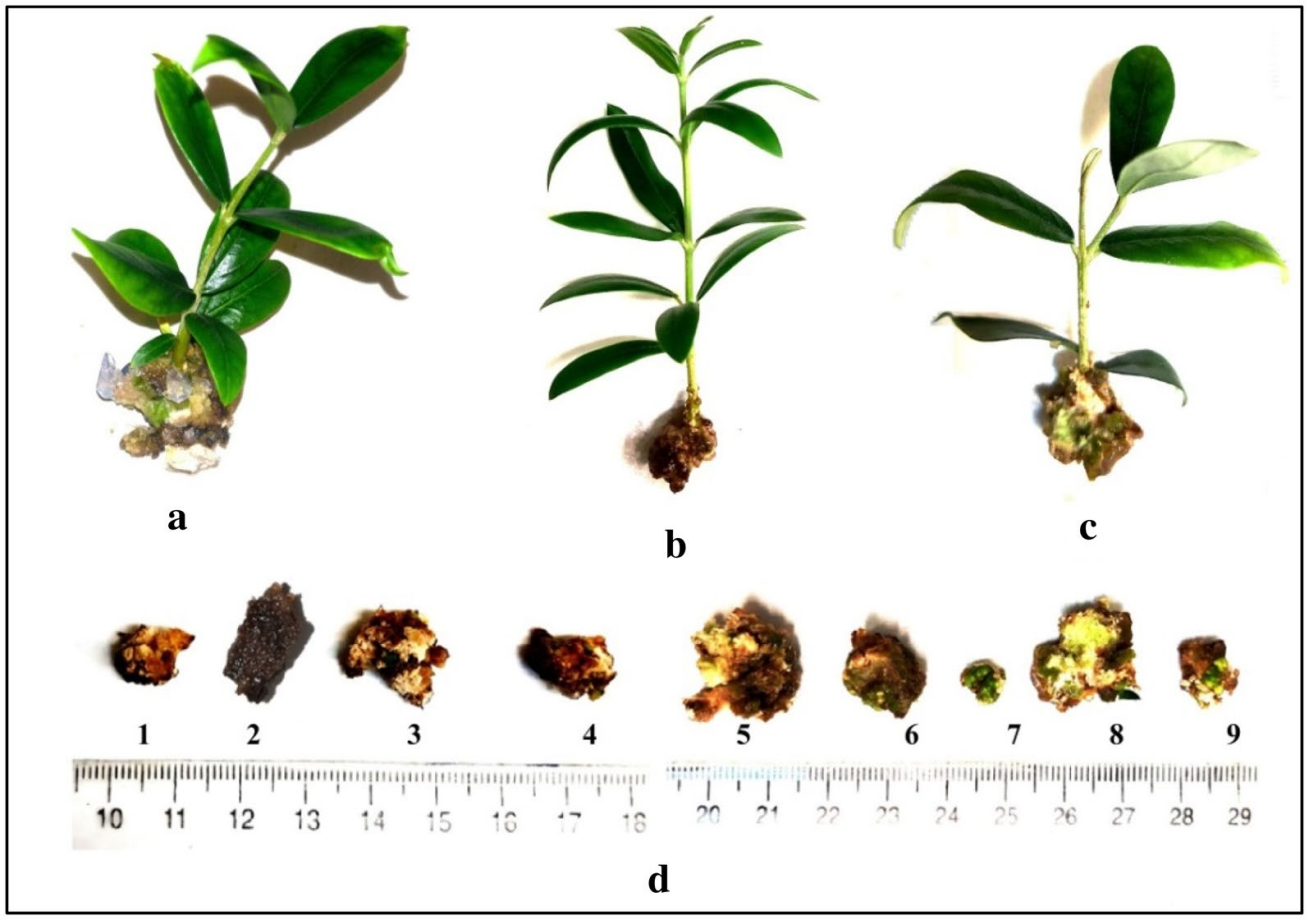
In vitro-raised **a** ˈMeshkatˈ, **b** ˈArbequinaˈ and **c** ˈAminˈ cultivars in optimized media (Table 1) after 6 weeks, **d** different kinds of calli formed in ˈMeshkatˈ, ˈArbequinaˈ and ˈAminˈ cultivars: 1) spongy, 2) dead crystalized, 3) puffy, 4) dense vitrified, 5) spongy bicolored, 6) green solid, 7) green dense, 8) greenish spongy and 9) strict tricolored

Calli obtained from different media and genotype had different morphological characteristics like color and texture. In some cases, calli were green in color and strict solid in texture resulting in indirect organogenesis and their callus-differentiated shoots were morphologically healthy, but some of them were crystallized, spongy and vitrified which never gave rise to indirect shoot formation (Fig. 4).

### Callus formation at proliferation stage

In ˈArbequinaˈ and ˈMeshkatˈ cultivars, there was a direct correlation between zeatin concentration and callus formation, but in ˈAminˈ there was no correlation in callus production (Table 3). In ˈArbequinaˈ and ˈAminˈ cultivars, callus production and volume were significantly higher in PMB than SSM. Regarding carbon source and light intensity in ˈArbequinaˈ cultivar, mannitol resulted in 100% callus formation and 2000 cd sr m^−2^ produced more callus than 5000 cd sr m^−2^ (Table 3).

### Molecular assessment of CIS and MIS

From 12 primer combinations tested, 259 bands (50 bp to 1000 bp) were analyzed in all tested cultivars consisting of MIS and CIS of which 238 bands were polymorphic. The polymorphism rate varied from 39.02% for EAGG-MGC to 100% for EAGC-MCAA. Each primer pair resulted in 28 to 55 markers with an average of 42 amplified fragments (Table 4).

**Table 4:** Primer combinations and polymorphism rate

Primer combination	Primer sequence (5′ to 3′)	Total number of bands	Number of polymorphic bands	Polymorphism rate (%)
EAGG-MGT	GACTGCGTACCAATTCAGG GATGAGTCCTGAGTAAGT	37	28	75.67
ECT-MGAG	GACTGCGTACCAATTCCT GATGAGTCCTGAGTAAGAG	45	42	93.33
ECT-MGC	GACTGCGTACCAATTCCT GATGAGTCCTGAGTAAGC	37	32	86.48
EAGC-MCAA	GACTGCGTACCAATTCAGC GATGAGTCCTGAGTAACAA	50	50	100
MGT-ETA	GATGAGTCCTGAGTAAGT GACTGCGTACCAATTCTA	33	27	81.81
MGC-ETA	GATGAGTCCTGAGTAAGC GACTGCGTACCAATTCTA	55	25	45.45
EGA-MCAT	GACTGCGTACCAATTCGA GATGAGTCCTGAGTAACAT	39	34	87.17
MCT-EAGG	GATGAGTCCTGAGTAACT GACTGCGTACCAATTCAGG	28	21	75
MCAG-EAT	GATGAGTCCTGAGTAAGA GACTGCGTACCAATTCAT	71	63	88.73
EAGG- MCAG	GACTGCGTACCAATTCAGG GATGAGTCCTGAGTAAGA	28	25	89.28
EAGG-MTTT	GACTGCGTACCAATTCAGG GATGAGTCCTGAGTAATTT	35	16	45.71
EAGG-MGC	GACTGCGTACCAATTCAGG GATGAGTCCTGAGTAAGC	41	16	39.02
Total/Average		499	379	75.95

### Genetic similarity

**Fig. 5:**
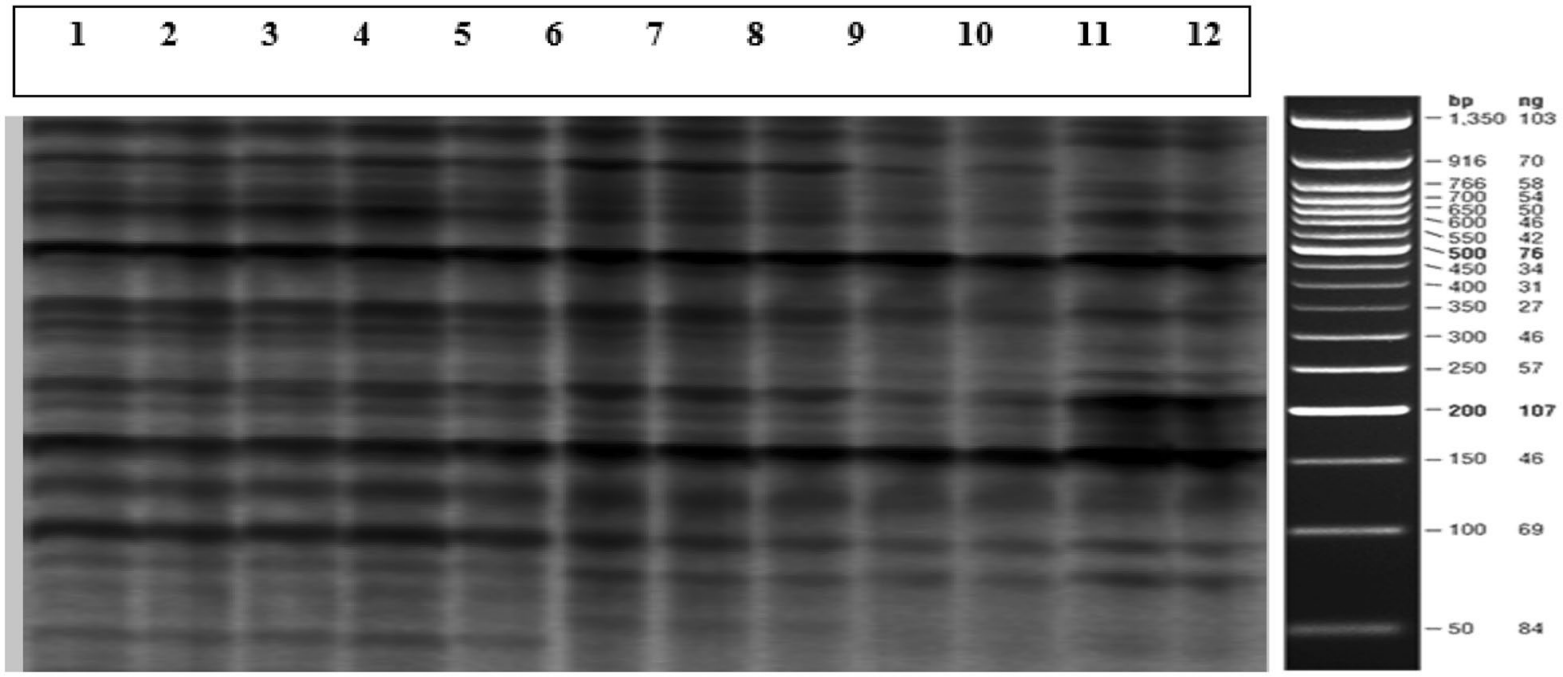
Highly monomorphic bands in MIS and CIS in 1) Meshkat4MIS, 2) Meshkat4CIS, 3) Meshkat2CIS, 4) Meshkat1MIS, 5) Arbequina2MIS, 6) Arbequina2CIS, 7) Arbequina3MIS, 8) Arbequina3CIS, 9) Amin3MIS, 10) Amin3CIS, 11) Amin4MIS and 12) Amin1CIS in application of EAGG and MGC primer pair (50 bp ladder, Fermentas, USA)

**Fig. 6:**
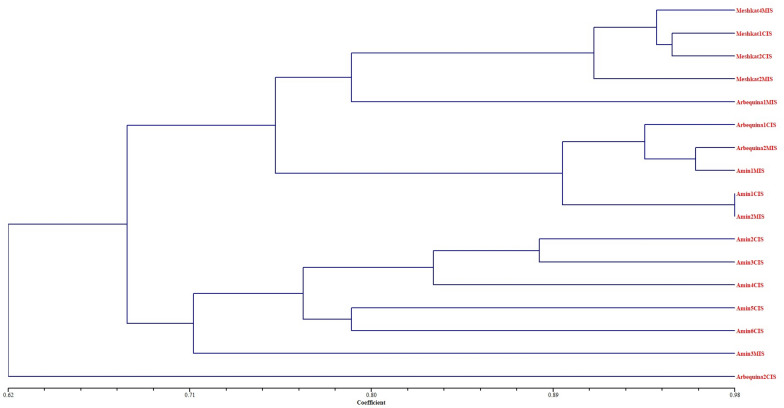
Dendrogram (NTSYS-PC ver. 2.02 package) of three cultivars with MIS and CIS

Clustering the CIS and MIS in ˈMeshkatˈ, ˈAminˈ and ˈArbequinaˈ cultivars was conducted according to the genetic similarity SAHN, method UPGMA and tie WARN. The phylogenetic analysis split the samples into 8 distinct groups indicating that callus-regenerated plants had highly monomorphic amplicons similar to those of the meristem-regenerated plants (Fig. 5). Cophenetic correlation coefficient (r) was 0.80 and based on Jaccard’s coefficient, similarity matrix and the pair-wise value between the CIS and MIS showed similarity levels at 62–98%. Marker analysis showed that genotype was the major factor in clustering the plantlets than the origin of propagation (Fig. 6). In ˈMeshkatˈ cultivar regardless of the origin of regeneration (meristem or callus), each sample had the highest resemblance with other samples according to the similarity coefficient factor, Jaccard and Dice (Fig. 6).

In ˈAminˈ cultivar, MIS and CIS were 98% similar in the third group. Also in ˈAminˈ MIS and ˈArbequinaˈ MIS more than 97% resemblance was seen. This group was the only group that consisted of both ˈAminˈ and ˈArbequinaˈ cultivars in both CIS and MIS type (Fig. 6).

One of the CIS in ˈArbequinaˈ cultivar appeared in a separate branch of phylogenetic tree which was similar to all groups consisting 3 cultivars with 62% genetic similarity and one of its MIS samples appeared in a separate category with 79% resemblance with both CIS and MIS of ˈMeshkatˈ. It is notable even in MIS samples for ˈAminˈ and ˈArbequinaˈ cultivars, more than 97% genetic similarity was obtained in the third category. In this study, ˈArbequinaˈ showed the highest dispersal (7–38%) in CIS and MIS, but both Iranian cultivars (ˈAminˈ (2–32%) and ˈMeshkatˈ (5–9%)) had more stability (Fig. 6).

### FCM assessment of CIS and MIS

**Fig. 7:**
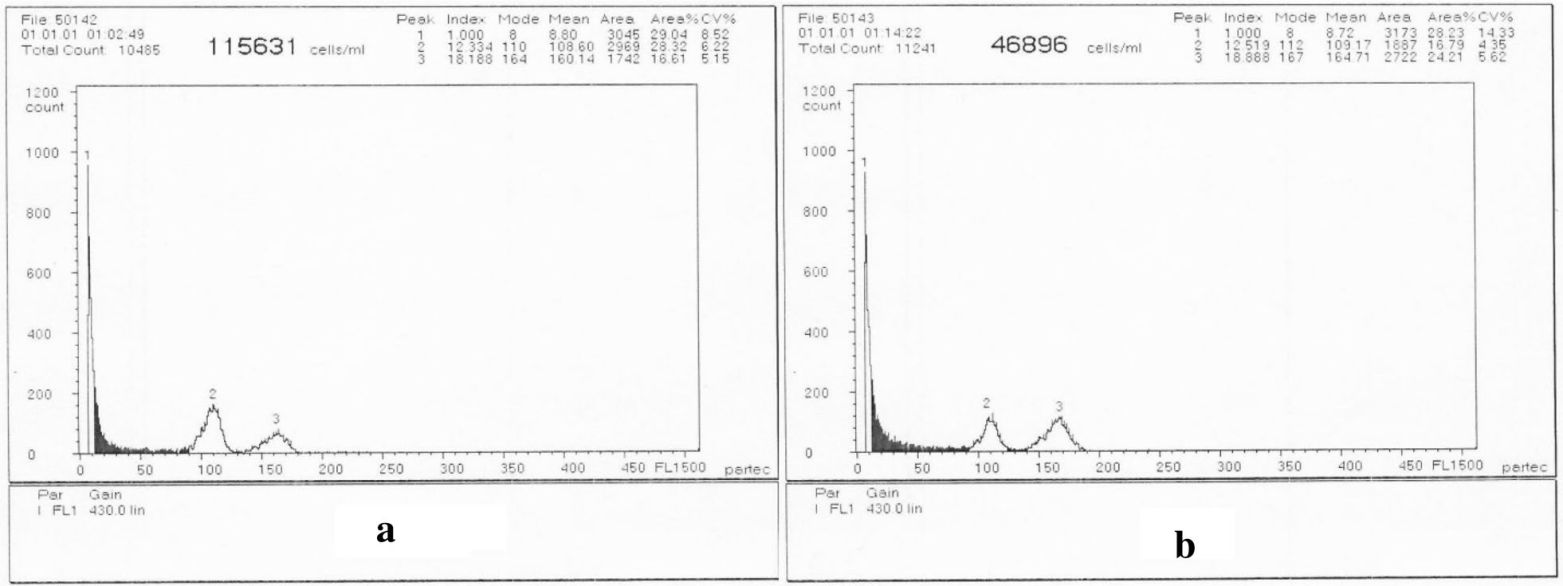
Typical histograms of ˈMeshkatˈ cultivar for **a** CIS and **b** MIS with more than 10,000 nuclei analyzed (mode P1/P2 = 0.67) with 3 repetitions

Simultaneous analysis of nuclei isolated from parsley *(Petroselinum crispum* Mill.*)* (2C DNA amount = 4.46 pg, as internal standard) and olive cv. ˈArbequinaˈ, ˈMeshkatˈ, ˈAminˈ in CIS and MIS resulted in highly reproducible histograms with low CV values indicating the priority of cultivar rather than the origin of propagation in genetic similarity (Table 5). There was some discrepancy (0.01–0.08 pg) between CIS and MIS in total DNA amount (2.87–3.12 pg) that varied among cultivars (Table 5). Figure 7 shows FCM histograms for CIS and MIS in ˈMeshkatˈ cultivar indicating the similarity between the peak_1_/peak_2_ ratios (mode P_1_/P_2_ = 0.67) in CIS and MIS.

**Table 5:** DNA amounts in ˈAminˈ, ˈMeshkatˈ and ˈArbequinaˈ cultivars regenerated from callus and meristem (mean ± SE)

Cultivar	Type of origin	P_1_/P_2_	DNA amount (pg)
ˈAminˈ	CIS	0.67 a	2.99 ± 0.01
	MIS	0.68 a	3.02 ± 0.01
ˈMeshkatˈ	CIS	0.67 a	3.01 ± 0.01
	MIS	0.64 a	2.87 ± 0.05
ˈArbequinaˈ	CIS	0.70 a	3.12 ± 0.02
	MIS	0.66 a	2.95 ± 0.03

## Discussion

### Effects of zeatin concentration and cultivar on growth indices at proliferation stage

Benelli and De Carlo [11] reported that zeatin (10 µM) not only affected axillary shoot induction of olive but also stimulated shoot length. Another study reported that 18.4 µM of zeatin caused a negative effect on olive growth indices [16]. Ali et al. [13] showed that 4 mg/L zeatin caused an acceptable microshoot formation of olive in comparison with 1, 2 and 3 mg/L. Zeatin is the best cytokinin enhancing shoot formation and even length development in olive influencing cell expansion. High zeatin concentration reduced shoot proliferation and elongation which are genotype-dependent [16]. Since supra-optimal concentration of cytokinins had a low interaction with minerals in the media, shoot length depression occurred in sub-optimal and supra-optimal concentrations [13].

It is showed that combined cytokinins in olive increased the proliferation rate [15], and so zeatin in combination with BAP, the constant PGR in this study, was used to exert a synergistic effect on proliferation improvement. OM supplemented with 3 µM zeatin and 0.5 µM BAP had the best effect on microshoot formation in ˈMoraioloˈ cultivar [13]. In other subspecies of olive (Laperrinei olive), regeneration was highly related to media and PGRs, and only in case of not applying PGRs, microshoot formation did not develop [16]. Zeatin and carbon source might have an effect on complex plant signaling network [19].

### Interactive effect of culturing system and genotype at proliferation stage

Apical dominance and oxidized tannins from phenolic compounds in semi-solid media inhibited the normal regeneration in olive [9, 10]. Using bioreactor is advantageous because of lower production cost, less labor, lower zeatin concentration and increased efficiency of proliferation rate as well as removing gelling agent [11]. A novel temporary immersion system is reported to be a suitable method in olive micropropagation in comparison with other liquid systems such as Erlenmeyer flasks, filter paper bridges in test tubes and LifeReactor [12]. The advantage of liquid system is due to lowering the toxic compound aggregation, well aerated plantlets [9, 11] and horizontal position of plantlets. This method allows reduction in carbon dioxide, ethylene gases and relative humidity in the vessel headspace and thus reduces morphogenesis abnormality. It is showed using plantform™ bioreactor, zeatin concentration in olive reduced to 25–50% [11]. However, Sadder [9] reported the suffocation of olive cultivar ˈNabaliˈ in liquid OM.

Analysis of olive shoot tip (4–5 cm) by Rugini [10] resulted in introducing the best medium for olive micropropagation called OM which is high in Ca, Mg, S, Cu and Zn elements. Mg which is triple higher in OM compared to MS and VS (Duchefa, NL) increased shoot elongation by positively affecting enzymes, especially those involved in phosphate transfer as modulator of shoot initiation and growth promoter [26]. It has as twice Boron as MS and VS which evidently plays important roles in nucleic acid synthesis, cell differentiation and elongation [27].

VS medium contains macro elements as described by Murashige and Skoog [28]. It also has chelated Fe form of EDDHA instead of EDTA as described by Van der Salm et al. [29] which provides a better iron supply at higher pH and phosphate content media. It is showed that OM in olive cv. ˈArbequinaˈ was more effective than woody plant medium (WPM) and MS in producing total number of shoots and healthy plantlets [15]. Different fundamental formulations are required for olive genotypes [10, 15], as minerals may sensitize cells to PGRs and nutrient mobilization occurs in PGRs existence by creating new source sink relationship. High calcium (CaCl_2_ and Ca(NO_3_)_2_) in OM plays an important role in cytokinin signal transduction in cells leading to better cell division [13]. Folic acid co-enzymes are involved in carbon transfer, which is vital for methionine, serine, deoxythimidylic acid and purines synthesis that are necessary for cell differentiation [13].

### Carbon and light sources

Leva et al. [19] showed more undifferentiated parenchyma-like callus growth in medium containing sucrose compared with mannitol. Haddad et al. [16] reported mannitol enrichment resulted in greener foliage and morphologically healthy plantlets similar to their mother plants. It is proved that shoot development pattern, plantlet survival, growth quality and secondary shoot formation in olive are affected by carbon sources [19]. Starch, mannitol and sucrose are the major carbohydrates in olive and mannitol is predominant in leaves and branches twice as abundant as sucrose [18]. Mannitol is a sugar alcohol consisting of 30% of total carbohydrates in olive [30], which varies according to the year of bearing (on or off year), season (as mannitol peaks is related to high temperature in summer), olive organs and harvesting time [18]. In olive, subsequent sub-culturing (seven times) in media containing mannitol increased the survival rate approximately twice compared with sucrose [19]. Mannitol promoted bud sprouting and secondary shoot uniformity rather than sucrose regardless of its concentration [19]. It may directly and indirectly influence endogenous hormonal status protecting against detrimental compounds in media [19] in contrast to sucrose in higher concentrations. As mannitol is involved in activating osmotic effect, plants with high amount of mannitol or well-acclimatized in mannitol enriched media have negligible osmotic stresses [15]. Moreover, mannitol transfer in intracellular metabolism aids olive against abiotic stresses by acting as a supportive agent against olive cell suspension dehydration [19]. In catabolism of mannitol in sink cells, hexose phosphate generates 2 ATP molecules, while initial generation of hexose phosphate in catabolism of sucrose spends ATP, therefore the energetic advantage of mannitol might be used for exponential growth [15, 19].

In stevia, shoot induction occurred more frequently at 6000 cd sr m^−2^ intensity rather than 2000 and 4000 cd sr m^−2^ under the controlled situation. Light intensity also influenced stevia organogenesis and 6000 cd sr m^−2^ was suitable for axillary shoot formation [31]. Three light intensities (500, 1000, 2000 cd sr m^−2^) were compared in *Ternstroemia gymnanthera* and the results showed 1000–2000 cd sr m^−2^ were more acceptable in growth quality and in case of LED light and fluorescent lamp, the best luminous intensity was 2000 cd sr m^−2^ [32] similar to this study findings.

Callus is normally regenerated due to zeatin application in the proximal end of plantlets and at the end of petioles which is a common reaction to in vitro stresses. It is reported that OM induced callus in olive without additional PGRs [9]. However, callus organogenesis only occurred in special olive cultivars such as ˈAminˈ, ˈMeshkatˈ and ˈArbequinaˈ cultivars in comparison with two recently introduced Iranian cultivars ˈDireˈ and ˈTokhm-e-Kabkiˈ (under submission data) and two commercial cultivars ˈConservoliaˈ and ˈKoroneikiˈ.

### Callus formation at proliferation stage

Krishna et al. [22] reported that making wound in cutting samples, PGRs like auxin and auxin analogs, and sterilization can cause mutagenesis as well as changing growing conditions such as salts, temperature and light can result in oxidative stresses. Consequently, superoxide, hydrogen peroxide, hydroxyl, peroxyl and alkoxyl compounds can cause hyper/hypo methylation of DNA, DNA base deletion and substitution, chromosomal rearrangement and number change, resulting in mutation and somaclonal variation of in vitro culture [22, 23].

### Molecular assessment of CIS and MIS in three cultivars

In order to assess the olive genome coverage, several primers should be tested to increase the number of loci. It is previously stated that phenotypical, cytological and biochemical methods can be used for this purpose, though molecular assessment repetition in another growth phase is desirable [33] as somatical genetic stability of olive is scarcely studied so far. SSR was not informative in olive genetic variation assessment since replication slippage was more frequent that might ignore single nucleotide mutation [33], and so AFLP is chosen because its discriminating capacity was proved in analyzing olive cultivars [25]. Since stem cutting in vitro culture in olive was stable, therefore 97% resemblance between ˈAminˈ MIS and ˈArbequinaˈ MIS proved high similarity between these cultivars [20, 23, 33]. Embryogenic culture-induced olives from the same cotyledon maintained in in vitro conditions for 3 years showed genetic instability due to the long term maintenance [33]. Although variation in olive was reported slightly higher in aged plants in vitro subcultured continually, it was highly genotype-dependent and biometric analysis of quantitative traits also showed intraclonal variation. Haddad et al. [16] reported all amplicons in ISSR marker in regenerated diploid and triploid endangered olive compared with donor plants were monomorph, and so all the regenerants in high PGRs and their mother plants were grouped in the same category. Since the stability of in vitro-raised olives in OM and zeatin enriched media is proved, MIS was considered as donor plants and CIS was compared to MIS in this study. In addition, Guillaume et al. [34] reported that meristem mutation position is the main important factor in chimerism maintenance and mutation in apical dome normally results in permanent chimera [34]. In marker investigations, DNA is usually extracted from leaves and so, the mutation level in diploids might be underestimated [34]. Nevertheless, sampling after 6 subcultures could resolve this problem as showed by the results of this study.

Similarly, no correlation between genetically somaclonal varied plants with phenotypic changes was found in this study as phenotypical characteristics are controlled by several genes [33]. It should be noted that the special part of this genuine variation could be due to the differences in non-coding portion of genome, which might not be expressed and revealed in olive appearance and performance. To reduce such controversial issues, complementary phenotypic analysis is recommended [23]. Provoked changes in cells might also result in developmental reprogramming in plants [25, 33]. Callus phase is a prerequisite of variations and the problem of undesired callus induction at the basal end and petiole end in olive propagation is frequently reported. Studies on genetic stability in somaclonal varied olives are scarce [33] and so, this is the first study that compared genetic stability and ploidy level fidelity in olive plantlets originated from MIS and CIS. Belaj et al. [25] stated that the combination of two different genetic change assessments in olive is another approach and so, in the present study, evaluation of ploidy level changes by FCM was investigated.

### FCM assessment of CIS and MIS

In this study, DNA amounts were similar to olives measured previously (2.90–3.07 pg) [7]. DNA amount fluctuation might be attributed to the cytosolic compounds like phenols causing tannic acid effect as a result of interference with DNA-PI staining since there is an abundant amount of such materials in woody plants [7]. These interfering phenolic compounds are genotype-dependent and normally micropropagated plants accumulate higher amounts of cytosolic compounds which do not allow proper PI staining resulting in lower DNA amount [7]. Olive is extremely high in the above-mentioned compounds and so, its discrepancy in 2C DNA value should be considered cautiously. PVP was added to nuclei isolation buffer to resolve such problematic issues in secondary metabolites [7]. Not severely chopped leaves by razor blade can also equilibrate interferer material [7].

Loureiro et al. [7] reported that 2C DNA content of *O. europaea* ssp. *europaea* var. *europaea* ranged between 2.90 ± 0.020 pg/2C and 3.07 ± 0.018 pg/2C and for wild olive, it was 3.19 ± 0.047 pg/2C. In this study, 84% of samples had lower DNA amounts which might be due to PI staining. Ploidy level changes are prominent processes in plant evolution and due to the reduction of inbreeding depression effect, triploids are much more vigorous than diploids especially in extreme situations. Because ˈAminˈ is highly resistance to salinity stress and other abiotic and biotic stresses (under submission data), it was suspected to be a polyploid. Although this was not proven in this study as previously reported for other Iranian olive cultivars, studying other genetic variation disorders in this genotype is recommended.

## Conclusion

Mass propagation of olive cultivars has several difficulties leading to genotype-dependent low micropropagation rate. The genetic stability of olive during in vitro culture is fundamental for the elite olive genotypes selection. In order to develop certified true-to-type plant materials, the genetic stability of microshoots propagated from callus and meristem of three olive cultivars was studied. The results showed the cultivars had their specific optimal media and zeatin concentration. The high zeatin concentration resulted in depression in ˈMeshkatˈ cultivar, while it increased the micropropagation rate in ˈAminˈ cultivar. Mannitol as carbon source and lower light intensity greatly enhanced ˈArbequinaˈ growth indices. PMB promoted the number of axillary shoots in ˈAminˈ, but decreaed the growth quality indices of ˈArbequinaˈ. This study indicated the normal co-production of larger calli under optimized protocols led to the undesired indirect organogenesis. The results showed DNA amount varied among CIS and MIS. In marker analysis, clustering happened with the first priority of cultivar, however maximum 38% discrepancy was observed in ˈArbequinaˈ MIS and CIS. ˈArbequinaˈ MIS showed the highest resemblance (97%) with ˈAminˈ MIS, which is a highly resistant native cultivar to salinity stress. This study indicated that ˈAminˈ, an oily cultivar and more dwarf than ˈArbequinaˈ, can be suitable for the establishment of modern commercial orchards. ˈAminˈ with more stability in micropropagation showed a good potential substitution for commercial ˈArbequinaˈ cultivar. However, the variations happened to CIS can be considered as an alternative technique to other biotechnological methods in olive breeding programs for future studies.

## Methods

### Plant materials and general procedures

Three commercial cultivars including one Spanish cultivar (ˈArbequinaˈ) and two native cultivars (ˈAminˈ and ˈMeshkatˈ) were selected. The huge number of semi-hard cuttings with apical and lateral buds consisting of axillary buds were collected in July from mature trees grown in olive collection at Tarom Olive Research Station (Tarom, Zanjan, Iran). Explants with at least 2 opposite nodes (2 cm) were surface sterilized in 70% ethanol (v/v) for 60–75 s followed by agitating in 2.5% sodium hypochlorite containing 1% Tween-20 for 13 min, then three times washing in sterilized distilled water each time for 5 min.

At establishment stage, a large number of explants were cultivated on OM (Olive medium, Duchefa, NL) supplemented with 4 mg/L zeatin (Duchefa, NL) and 0.5 mg/L 2iP (6-γ γ-Dimethylallylaminopurine) (Duchefa, NL) in order to have enough sources for experiments.

All media including solid and liquid were enriched with 3% (w/v) sucrose (unless otherwise stated) and 0.5 mg/L 2iP + 0.23 mg/L BAP + 0.04 mg/L IBA + 540 mg/L calcium gluconate + 50 mg/L FeEDDHA + 4 mg/L H_3_BO_3_ and pH was adjusted to 5.8 ± 0.2. They were solidified with 0.7% (w/v) plant agar (Duchefa, NL). All media and glassware were autoclaved for 15 min at 121 °C. Phytotrons with high pressure metal halide lamps (2609 cd sr m^−2^) and 16/8 h light/dark cycle and temperature of 24 ± 1 °C were used to maintain the cultures. Olive growth indices, namely number of axillary shoots, leaves number, shoot length and callusing were measured and recorded in all experiments. At least 25 repetitions of each treatment were averaged.

### Interactive effects of cultivar, culture media and zeatin concentration at proliferation stage

Three different olive cultivars (ˈArbequinaˈ, ˈAminˈ and ˈMeshkatˈ), two culture media (OM and MS) [28] and various zeatin concentrations (0, 1, 2 and 4 mg/L) were added to basal studied media (plant materials and general procedures).

Application of Periodical Mini Bioreactor (PMB) at proliferation stage.

Two cultivars (ˈAminˈ and ˈArbequinaˈ) under different culture media (OM, MS and VS) [29] containing 2 mg/L zeatin beside the previously mentioned additives (plant materials and general procedures) in two forms of semi-solid media (SSM) and liquid were investigated. VS was included in this experiment, because in our previous study olive cv. Amin was reacted well to it [35].

**Fig. 8:**
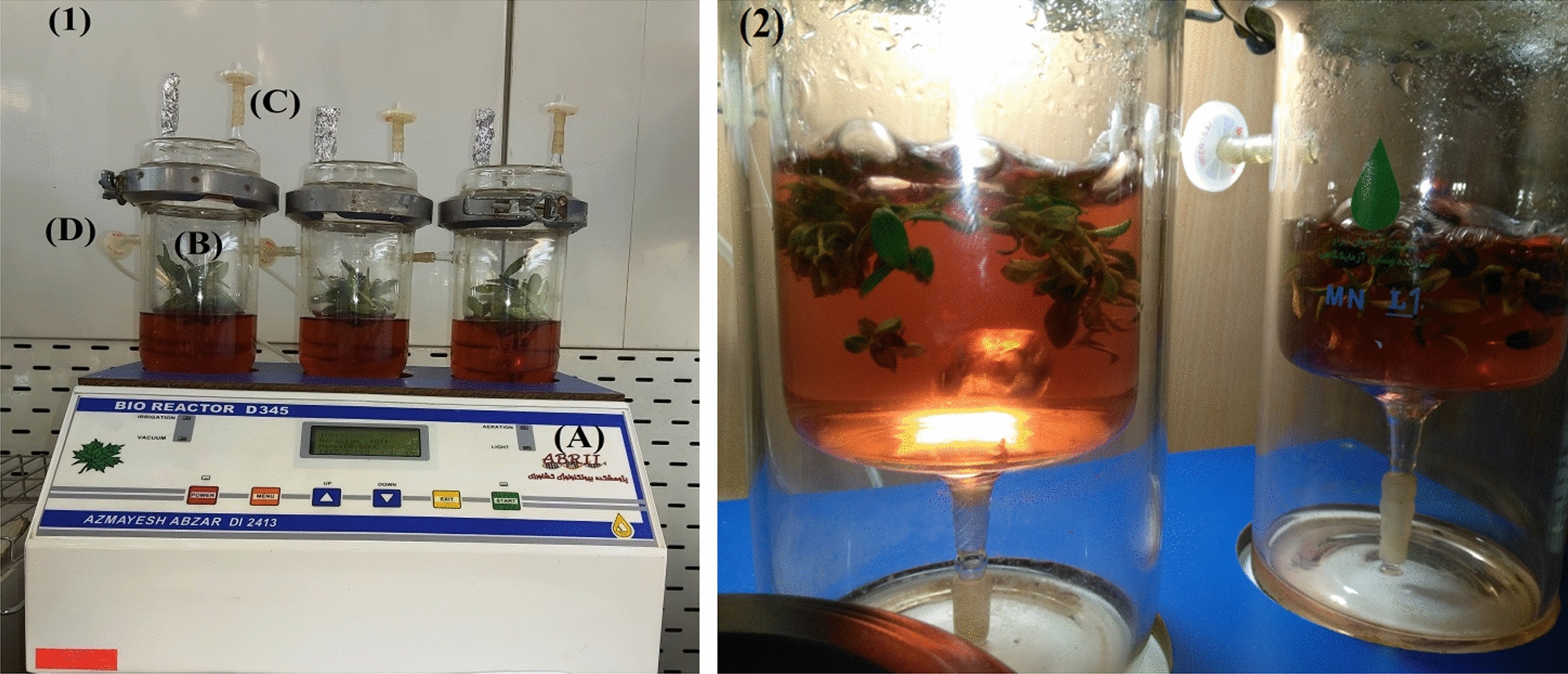
Periodical mini bioreactor (PMB) system: (1) complete equipment including **a** controller, **b** jars with two glass layers which inner layer contained the plant material and outer layer contained the culture medium, (**c**) and (**d**) 0.2 µm cellulose nitrate filters for entrance of sterilized air to equipment (where (**c**) was for vacuuming air, and (**d**) was for importing air and vice versa) (2) immersion step

Autoclaved periodical mini bioreactors (BPM; bioreactor D 345, AZMAYESH ABZAR DI 2413, IR) were filled with 300 mL of sterilized liquid medium (Fig. 8). Ten explants with three replications were placed in the inner jars and periodically immersed in the liquid medium. The compressed air passed through two 0.2 µm micropore filters providing additional protection. The nutrition program in 24 h included three times immersion (each time 10 min) followed by three times drainage and aeration (each time 10 min) and stationary stage (each time 7 h and 40 min).

### Effect of carbon source and light intensity at proliferation stage

To optimize proliferation stage, two carbon sources (sucrose and mannitol) and two different light intensities (2000 and 5000 cd sr m^−2^) in OM containing 1 mg/L zeatin with previously mentioned additives (plant materials and general procedures) for 25 samples (5 glass jars and 5 sample per each) were studied.

### Genetic stability in callus induced shoots (CIS) and meristem induced shoots (MIS)

Since large masses of calli at the basal part of shoot explants in most olive proliferation experiments were created, callus formation percentage and callus volume (cm^3^) were recorded. For volume measurement, sphere volume (4/3 π r^3^) for approximately spheral calli and immersion in graduated cylinder half filled with water for multi-dimensional calli were used. To confirm the similarity of microshoots originated from calli (CIS) and shoots originated from meristems (MIS), Amplified Fragment Length Polymorphism (AFLP) and Flow cytometry (FCM) were used. The shoots were cultured on their optimized media (Table 1) for 6 months while subcultured every 6 weeks. During this experiment, CIS samples equal to their counterpart MIS were marked and subcultured separately in order to check their similarity. Repetitions are mentioned in each experiment.

### Amplified Fragments Length Polymorphism (AFLP)

Total genomic DNA was extracted from 200 mg of fresh tissue of in vitro plant leaves with 2–6 repetitions and two sub-repetitions of both MIS and CIS following the Cetyl Trimethyl Ammonium Bromide (CTAB) method [36]. DNA concentration and quality were evaluated by 1% agarose gel electrophoresis and NanoDrop (Implen, DE). The AFLP technique was carried out as described by Vos et al. [37] with the following modifications: 500 ng of genomic DNA of samples were double digested using 5 units of EcoRI and MseI enzymes, and adaptors were ligated to the obtained fragments. The ligation mixture was diluted 1:4 with nuclease-free water, and 3.75 µL of it was used for PCR pre-amplification in a 25 µL volume with 10 picomol of each pre-amplification primer carrying three selective nucleotides. After 2 min at 72 °C, 20 cycles were carried out at 95 °C for 30 s, 55 °C for 30 s and 72 °C for 60 s in Thermocycler (Applied Biosystems, USA). A total of 12 primer combinations of M and E primers with selective nucleotides were screened among those previously tested in the lab because they provided the highest number of heterozygous bands in the literatures as well. E primers were end-labeled radioactively with IRD700 and IRD800. The amplified products were separated by denaturing for 6 min at 96 °C on 6% polyacrylamide gel done at 1500 V, 60 mA, and 15 W for 60–90 min at 55 °C by DNA analyzer 2400 (Bioscience, USA). Amplified bands were scored as present (1) or absent (0), smeared and weak bands were discarded. Only reproducible bands were used to construct the original binary data matrix. Genetic similarities among accessions were evaluated by estimating Dice and Jaccard methods [38, 39]. The generated dendrogram (NTSYS-PC ver. 2.02 package) was compared by computing the cophenetic correlation. Genetic similarity (GS) between two cultivars i and j was calculated based on Jaccard’s similarity coefficient [39], GS ij = *a* / (*n*—*d*), where *a* is the number of fragments in common between the two cultivars, *d* is the number of fragments absent in both cultivars and *n* is the total number of fragments scored.

### Flow cytometry (FCM)

Young leaf segments (0.5 cm^2^) from 6 samples (with 3 repetitions) of the actively growing in vitro olive shoots and the same size of fresh young leaf of parsley (*Petroselinum crispum* Mill.) (2n = 2x = 22; 2C DNA amount = 4.46 pg, as an internal standard), were chopped with a razor blade in 400 µL nuclei isolation buffer (Partec, Münster, Germany) on a plastic petri dish according to Yokoya et al. [40]. To reduce the interaction between secondary compounds, 10 g/L polyvinyl pyrrolidone (PVP) was added to the nuclei isolation buffer. The lysate was filtered through a 50 μm Cell Trics and brought up to 2 mL with staining buffer (Partec) into which 12 μL Propidium Iodide (PI) and 6 µL ribonuclease (RNase) was added. In order to eliminate cell debris, the nuclear suspension was then filtered through a 30 μm Cell Trics. After 5 min incubation period on ice, around 10,000 nuclei were analyzed by FCM (PA2, Partec). Fluorescence intensity (540 nm and 615 nm for excitation and emission wavelengths, respectively) was measured by means of laser beam. Estimates of the ratio of fluorescence intensities of each olive to parsley sample ratio (P_1_/P_2_) were based on the means of at least 3 repetitions for each plantlet and 10 replicate for either CIS or MIS of each genotype. The peaks with a coefficient of variation of less than 5% were accepted and the nuclear genome size of each type of plant material was estimated according to Yokoya et al. (2000).

### Experimental design and statistical analysis

**Fig. 9:**
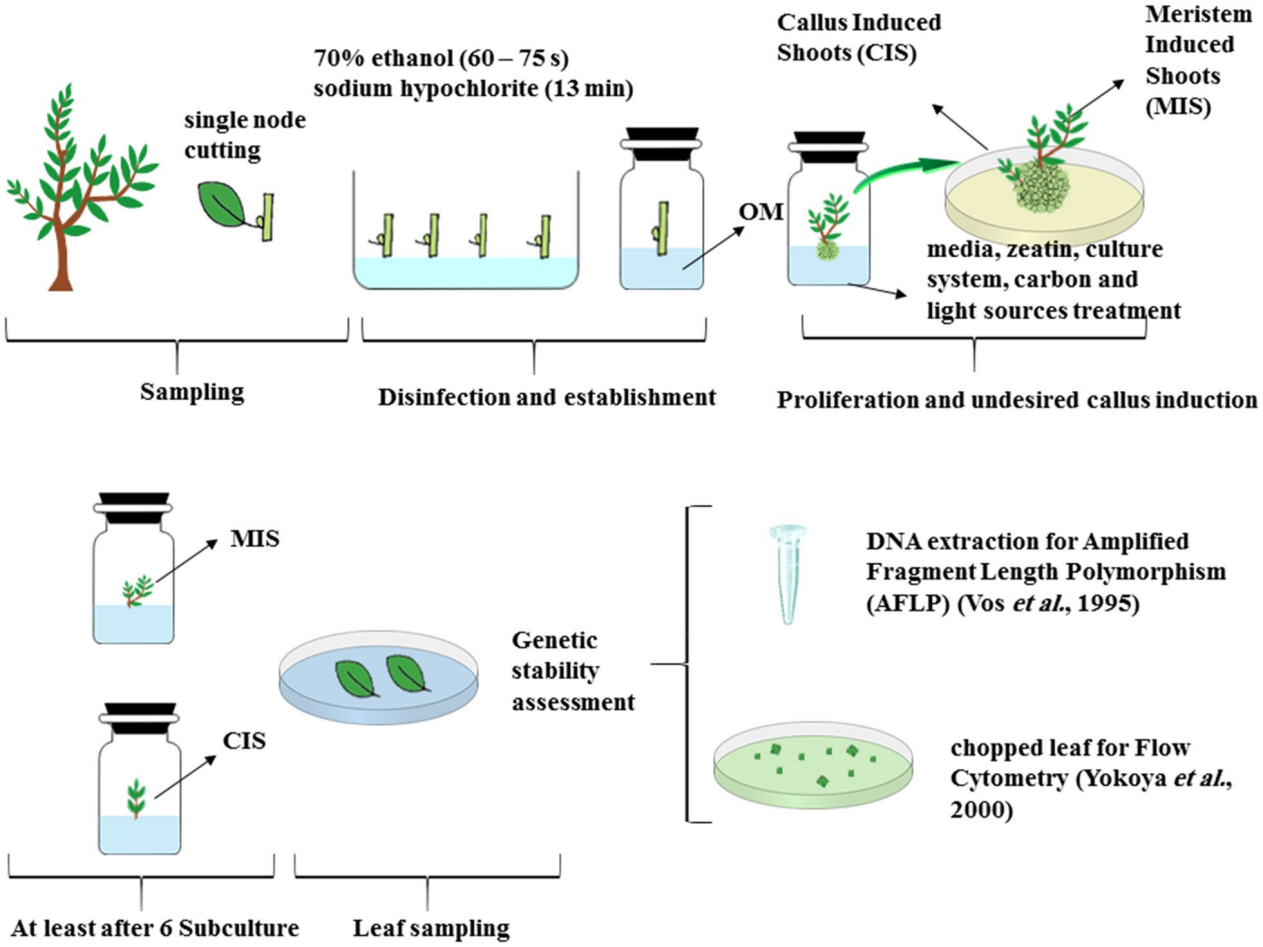
Schematic flow diagram of olive (*O. europaea* L*.*) ˈAminˈ, ˈArbequinaˈ and ˈMeshkatˈ micropropagation: well-grown single node cuttings were surface-sterilized and established in modified OM (OM supplemented with 4 mg/L zeatin and 0.5 mg/L 2iP). During proliferation stage, regarding the media with unintentionally callus formation (high zeatin media, mannitol enriched media and lower light intensity condition), CIS and MIS were proliferated separately. In vitro plantlets were subcultured for at least 126 days. Then leaf materials were tested for genetic stability assessment by AFLP and FCM

A flow diagram of the experimental design is illustrated in Fig. 9. It shows the processes of sampling, formation of CIS and MIS and leaf sampling for assessment of genetic diversity. The diagram simply shows the materials and methods highlighting undesired callus formation at the base of plantlets to distinguish shoots originated from callus and those generated from meristem (normal in vitro proliferation), which necessitates the investigation of genetic stability of CIS and MIS.

The micropropagation experiments were conducted under a completely randomized design with at least 25 replications. In all proliferation experiments, growth indices including number of axillary shoots, main shoot length (cm), number of leaves on main shoot and visual growth quality (1–5 scoring where 1 and 5 were the minimum and the maximum qualities, respectively) were recorded after 6 weeks. The percentage of callus production at the base of in vitro shoots and callus volume (cm^3^) were also recorded. Data were evaluated by the analysis of variance (ANOVA) according to the general linear model (GLM) procedure using statistical software SAS (Institute Inc., Cary, NC, USA). Mean comparisons among treatments were conducted using LSD at the *P* ≤ *0.01* level of probability.

## Data Availability

All data generated or analyzed during this study are included in this published article and its supplementary information files or the datasets used and/or analyzed during the current study are available from the corresponding author on reasonable request.

## Abbreviations

AFLP: Amplified fragments length polymorphism
BAP: 6-Benzylaminopurine
CIS: Callus-induced shoots
FCM: Flow cytometry
MIS: Meristem-induced shoots
MS: Murashige and Skoog;
OM: Olive medium (ROM, Rugini olive medium)
WPM: Woody plant medium
PGRs: Plant growth regulators
PMB: Periodical mini bioreactor
PVP: Polyvinyl pyrrolidone
VS: Van der salm
2iP: 6-γ γ-Dimethylallylaminopurine
